# The immunological paradox: immune checkpoint inhibitors in liver transplantation

**DOI:** 10.1007/s10147-026-03091-2

**Published:** 2026-07-11

**Authors:** Takashi Ito, Shinya Okumura, Katsunori Sakamoto, Etsuro Hatano

**Affiliations:** Department of Surgery, Graduate School of Medicine, Kyoto University, 54 Kawahara-Cho, Shogoin, Sakyo-Ku, Kyoto 606-8507 Japan

**Keywords:** Liver transplantation, Immune checkpoint inhibitor, T cell mediated rejection, Hepatocellular carcinoma, Perihilar cholangiocarcinoma

## Abstract

The incorporation of immune checkpoint inhibitors (ICIs) into the treatment repertoire for hepatocellular carcinoma (HCC) and advanced biliary tract cancers has significantly transformed the field of oncology, offering remarkable survival advantages. However, the interaction of these powerful immunomodulators with liver transplantation (LT), the ultimate curative intervention for end-stage liver disease, presents a complex immunological paradox. While LT depends on the induction and maintenance of immune tolerance to suppress alloimmune responses and prevent allograft rejection, ICIs operate by disrupting these tolerance mechanisms to enhance host antitumor immunity. This comprehensive review synthesizes the latest evidence from 2024 and 2025, incorporating key findings from the VITALITY study, international washout cohorts, and meta-analyses of individual patient data. The historical "Liver Tolerance Effect," the mechanistic role of the PD-1/PD-L1 axis as a crucial protector of hepatic integrity, and the severe phenomenon of ICI-induced fatal hepatic necrosis are critically examined in this review. Furthermore, the emerging utility of precision predictive biomarkers, including immune-related adverse events (irAEs), the Eplet Risk Score, and intragraft PD-L1 expression as tissue-based predictors of post-transplant ICI-induced rejection, was rigorously analyzed to stratify rejection risk. By examining the pharmacodynamic conflict between systemic tumor eradication and localized graft preservation, this report provides a pragmatic, evidence-based framework for candidate selection, optimal washout timing, and post-transplant management, ultimately culminating in a critical appraisal of the ethical imperatives surrounding living donor liver transplantation.

## Introduction: the collision of two therapeutic paradigms

The current clinical management of hepatocellular carcinoma is characterized by the intersection of two distinct, advanced, and immunologically opposing medical advancements. Over the past four decades, liver transplantation has evolved from an experimental surgical procedure into a highly effective, mainstream curative treatment for patients with underlying cirrhosis and early stage morphology-limited tumor burdens. The sustained success of this surgical approach is entirely dependent on the pharmacological capacity to precisely control and suppress the recipient’s immune system, thereby preventing it from recognizing, attacking, and ultimately destroying the non-self allograft [1–3].

In contrast, the field of systemic oncology has undergone a profound transformation owing to the extensive implementation of immune checkpoint inhibitors. These monoclonal agents specifically target coinhibitory receptors, most notably Programmed cell death-1 (PD-1), Programmed death-ligand 1 (PD-L1), and Cytotoxic T-lymphocyte-associated antigen-4 (CTLA-4), effectively "releasing the brakes" on the host immune system. This targeted pharmacological blockade facilitates the reactivation of exhausted or anergic T-cells, enabling them to recognize and aggressively eliminate malignant cellular populations [4–6].

Within the rapidly evolving field of "Transplant Oncology," there is significant and growing clinical interest in integrating these two potent modalities. The remarkable objective response rates achieved by contemporary combination immune checkpoint inhibitor (ICI)-based regimens have substantially driven their application in “bridging” patients on transplant waiting lists or in “downstaging” patients with locally advanced tumor burdens to align them with acceptable eligibility criteria. However, this clinical approach is associated with considerable physiological risks. The specific mechanisms of systemic immune activation necessary for effective tumor clearance pose a significant threat to the liver graft, whose long-term viability is entirely dependent on the sustained suppression of T-cell responses [6, 7].

As shown in Table 1, navigating this intricate landscape requires a multiphasic approach. Clinicians must carefully balance pre-transplant washout strategies against the significant risks associated with post-transplant salvage therapy, which are further informed by the emerging predictive biomarkers. The administration of ICIs before transplantation can effectively reduce the tumor burden; however, residual pharmacological activity at the time of reperfusion may precipitate hyperacute alloimmune rejection. This review delineates a comprehensive roadmap for clinicians operating at the critical intersection of transplant surgery and immuno-oncology. Literature Search Strategy: This manuscript constitutes a narrative literature review. A comprehensive search of PubMed/MEDLINE and the Cochrane Library was conducted for studies published betweJanary 2017 aMarrch 2025. The primary search terms included: “liver transplantation,” “hepatocellular carcinoma,” “immune checkpoint inhibitor,” “PD-1,” “PD-L1,” “CTLA-4,” “allograft rejection,” “washout period,” “transplant oncology,” and “living donor liver transplantation.” Studies were selected based on clinical relevance and methodological rigor, with priority given to prospective trials, large retrospective cohorts, and systematic reviews with meta-analyses. We acknowledge that this narrative approach is subject to selection bias, which represents an inherent limitation of this study.

**Table 1:**
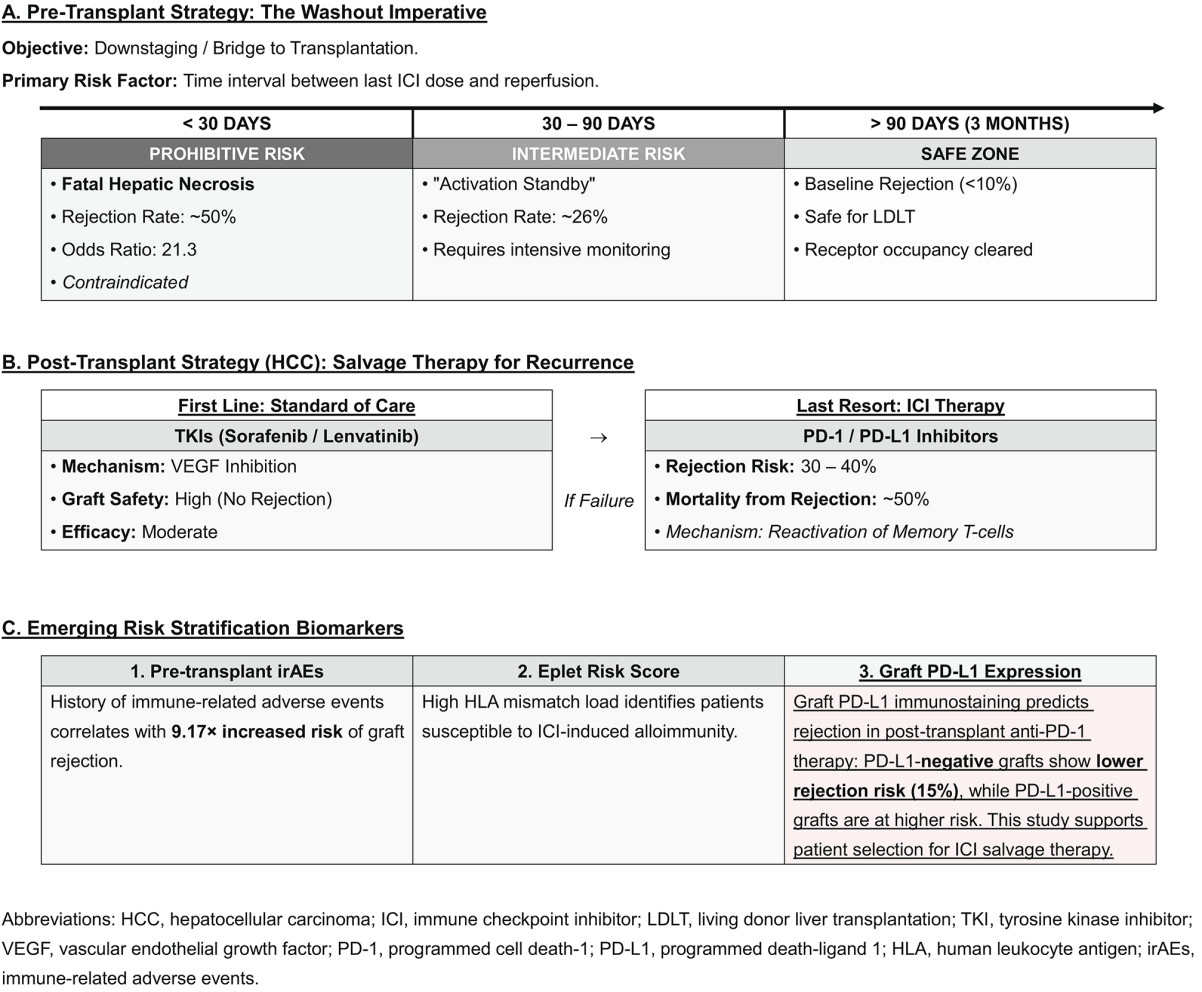
The strategy of immune checkpoint inhibitors use in liver transplantation

## The unique immunological status of the liver and checkpoint pathways

To fully understand the significant risks associated with perioperative ICI administration, it is essential to recognize the liver’s unique evolutionary status as an “immunologically privileged” organ.

### The liver tolerance effect and microchimerism

The liver exhibits unique tolerogenic characteristics that are unparalleled by those of any other solid organ. This phenomenon was first systematically documented in the 1960s by pioneering transplant surgeon Sir Roy Calne. Through a series of seminal surgical experiments, Calne observed that orthotopic liver allografts conducted in completely outbred major histocompatibility complex (MHC)-mismatched pigs could achieve long-term survival without the administration of exogenous immunosuppressive drugs [8, 9].

Thomas Starzl provided a pivotal mechanistic understanding of the "Liver Tolerance Effect" by demonstrating that successful graft acceptance is closely associated with "microchimerism." Starzl elucidated that donor passenger leukocytes actively migrate from the transplanted hepatic parenchyma into the recipient’s systemic circulation, thereby establishing a state of mutual non-reactivity. This process effectively educates the host immune system to accept the graft [10, 11] (Table 2).

**Table 2:**
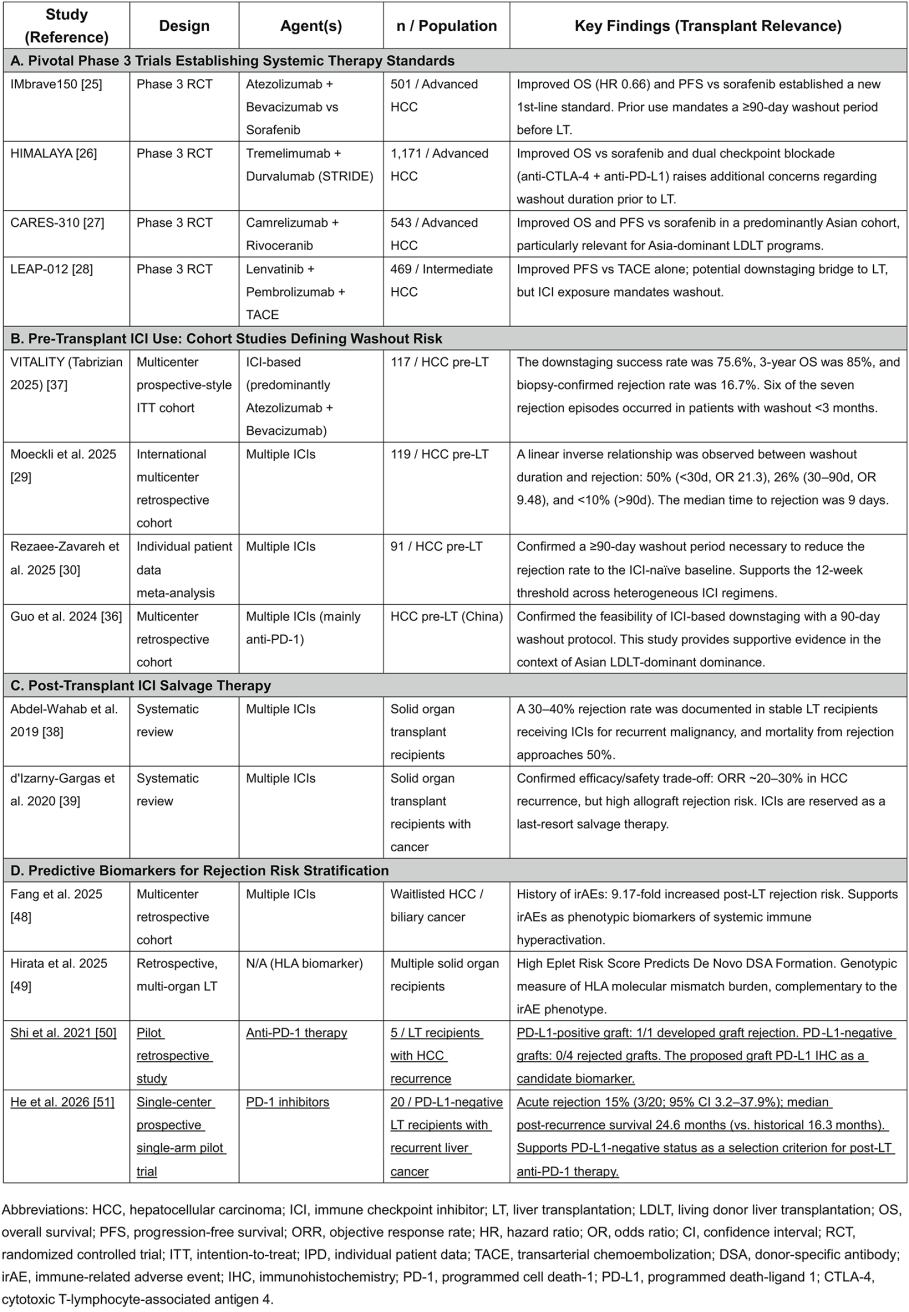
Summary of key clinical trials and cohort studies referenced in this review

### The PD-1/PD-L1 pathway: guardian of peripheral tolerance

While leukocyte microchimerism accounts for a substantial aspect of the liver’s tolerogenic properties, active maintenance of this state necessitates ongoing molecular regulation. The primary molecular mechanism underlying this physiological suppression is the PD-1/PD-L1 axis. Liver non-parenchymal cells, particularly Liver Sinusoidal Endothelial Cells (LSECs) and Kupffer cells, inherently express exceptionally high baseline levels of PD-L1. Upon entry of activated T-cells into the liver, the interaction between PD-L1 and the PD-1 receptor conveys a potent negative intracellular signal, leading to T-cell apoptosis or anergy. This “peripheral deletion” system persistently neutralizes potentially autoreactive or alloreactive T-cells [12–14].

### The pharmacological conflict: the "activation standby state"

Current immune checkpoint inhibitors (ICIs) are meticulously designed to disrupt these specific protective molecular interactions. By fully inhibiting the PD-1/PD-L1 pathway, ICIs effectively dismantle the liver’s primary defense mechanism against alloimmunity. The Japanese scientific literature characterizes this resultant condition as placing the recipient’s immune system in an “activation standby state,” wherein the immune system is effectively primed, expanded, and prepared to respond—a condition in which ICI-treated T-cells are reactivated and expanded in vivo but are held in a primed, ready-to-respond configuration. Upon antigenic stimulation at the time of allograft reperfusion, these cells can rapidly mount a vigorous and often irreversible alloimmune attack without the usual lag phase required for naïve T-cell priming. [13–16].

## Evolution of hepatocellular carcinoma treatment and transplant criteria

The rapid expansion of the criteria for transplant eligibility, alongside advancements in systemic oncological therapies, has inevitably led to their current convergence.

### From Milan to extended biological metrics

The criteria for determining the eligibility for liver transplantation in patients with hepatocellular carcinoma (HCC) have transitioned from rigid morphological constraints to more nuanced biological evaluations. The original Milan Criteria, established in 1996, limited eligibility to patients with a single tumor measuring ≤ 5 cm or up to three distinct tumors, each measuring ≤ 3 cm.1 To broaden access, a series of expanded criteria have been developed, including the UCSF and Up-to-seven Criteria [17–19].

In contemporary transplant oncology, there has been a significant shift towards the integration of dynamic biological markers, exemplified by the Toronto Criteria, which emphasize tumor differentiation. Within the realm of living donor liver transplantation (LDLT), the 5–5-500 rule permits transplantation for patients presenting with ≤ 5 tumors, each measuring ≤ 5 cm, on the condition that the alpha-fetoprotein (AFP) level remains strictly ≤ 500 ng/mL. This biologically informed approach has been independently validated using the AFP Model [20–22].

### The systemic therapy revolution

In parallel developments, the landscape of systemic medical therapy for advanced hepatocellular carcinoma (HCC) has transitioned from the use of relatively less potent tyrosine kinase inhibitors (TKIs), such as Sorafenib and Lenvatinib, to more efficacious immunotherapy approaches. The pivotal IMbrave150 trial demonstrated that the combination of Atezolizumab and Bevacizumab significantly surpassed sorafenib in terms of efficacy [43]. This advancement was promptly succeeded by the HIMALAYA trial, which validated the STRIDE regimen, as well as the CARES-310 and LEAP-012 trials, which illustrated that the addition of Pembrolizumab and Lenvatinib to Transarterial Chemoembolization (TACE) markedly enhanced progression-free survival. The notably high objective response rates achieved by these therapeutic combinations render them highly attractive options for the downstaging of aggressively proliferating tumors [23–28]. A structured summary of these pivotal Phase 3 trials, together with the major pre-transplant cohort studies (Sect.  "Pre-transplant Checkpoint Inhibition: Bridging, Downstaging, and the Washout Imperative"), post-transplant salvage studies (Sect. "Disease-Specific Modalities and Expanding Indications"), and predictive biomarker studies (Sect. "Emerging Risk Stratification Biomarkers"), is presented in Pre-transplant.

## Pre-Transplant checkpoint inhibition: bridging, downstaging, and the washout imperative

The application of immune checkpoint inhibitors (ICIs) as a strategic “bridge” or “downstaging” method prior to transplantation represents a highly contested area of research [29–42]. The primary variable under the control of clinicians is the "washout period," which refers to the interval between the final administration of ICIs and transplantation. Evidence emerging in 2024 and 2025 will transition from anecdotal case reports to robust quantitative data, establishing a clear correlation between the duration of this interval and the risk of rejection.

### Quantifying the safety window: the pharmacodynamic washout

As shown in Table 1, the primary variable under direct clinician control was the washout period. A seminal international retrospective study by Moeckli et al. provided the most comprehensive risk stratification to date. By examining 119 patients with HCC, the researchers established a perfectly linear inverse relationship between the duration of the washout interval and the probability of developing acute cellular rejection [29].

- Critical Danger Zone (< 30 Days): Patients who underwent transplantation within 30 days experienced a remarkably high rejection rate of 50.0%, with an adjusted Odds Ratio (OR) of 21.3.
- Intermediate Risk (30–90 days): Despite a moderate washout period, the rejection rate remained unacceptably elevated at approximately 26.0% (OR 9.48), necessitating intensive monitoring as the immune system remained in an “Activation Standby” state.
- Safe Zone (> 90 Days / 3 Months): Postoperative rejection rates only stabilized to standard baseline levels (< 10%) when the washout interval exceeded 90 days, thereby allowing for the clearance of receptor occupancy.

Importantly, the study observed that rejection episodes in the short-washout group occurred at a notably early stage, with a median of 9 days post-transplant.26 This timing lends support to the “activation standby” hypothesis, suggesting that the immune system is primed to initiate an immediate attack upon reperfusion, often preceding the establishment of therapeutic levels of calcineurin inhibitors, such as tacrolimus [29]. In support of these findings, an individual patient data (IPD) meta-analysis conducted by Rezaee-Zavareh et al. examined 91 patients with [30]. This study corroborated that a washout period of 3 months (approximately 90 days) is statistically necessary to reduce the likelihood of rejection to levels comparable to those in a population not exposed to immune checkpoint inhibitors (ICI). The convergence of data from various methodologies reinforces the 90-day rule as a crucial safety criterion. The discrepancy between the drug’s half-life (typically 2–3 weeks) and the required washout period (12 weeks) is explained by pharmacodynamics. Although antibodies may be cleared from the serum, receptor occupancy on T-cells can persist for several months.

### The clinical phenotype: fatal hepatic Necrosis

ICI-associated rejection is phenotypically distinct and significantly lethal. Nordness et al. documented a defining case of "fatal hepatic necrosis," wherein a patient treated with nivolumab underwent transplantation eight days following their final infusion [31]. The patient experienced fulminant hepatic necrosis, characterized by a substantial increase in CD8 + cytotoxic T-cells, which proved entirely unresponsive to high-dose steroid pulses and anti-thymocyte globulin (ATG), ultimately resulting in the patient’s death. Separate reports by Schnickel et al. and Dehghan et al. described catastrophic instances in which severe immune-mediated rejection led to the destruction of primary grafts, necessitating emergency rescue re-transplantation [32, 33]. Friend et al. similarly reported fatal rejections in the pediatric population [34]. Large cohort validations have confirmed that abbreviated washouts constitute the most significant independent risk factors for rejection and graft loss.

### Efficacy meets safety: the VITALITY study

Advancing beyond retrospective safety indicators, the VITALITY study conducted by Tabrizian et al. offers the inaugural prospective-style intention-to-treat (ITT) evidence regarding the efficacy of this therapeutic strategy [37]. This investigation, conducted across multiple centers in the United States, included 117 patients diagnosed with hepatocellular carcinoma (HCC). Significantly, 73.5% of these patients initially surpassed the Milan Criteria, indicating a cohort with restricted curative options. Following therapy based on immune checkpoint inhibitors (predominantly atezolizumab combined with bevacizumab), the study reported a notable 75.6% success rate in downstaging patients to meet transplant criteria.

Regarding survival outcomes, the 3-year post-transplant survival rate was 85%, which is comparable to traditional transplant results for HCC, thereby affirming the oncological efficacy of this approach. Nevertheless, consistent with previously described washout data, biopsy-confirmed rejection was observed in 16.7% of transplanted patients in this study. Importantly, six of the seven rejection episodes occurred in patients who experienced a washout period of less than 3 months, underscoring the 90-day rule as a crucial safety threshold for preventing graft loss while preserving oncological benefits [37].

### Mechanistic nuances and consensus recommendations

An emerging hypothesis within the field posits a potential differential safety profile between PD-1 inhibitors (e.g., Nivolumab, Pembrolizumab) and PD-L1 inhibitors (e.g., Atezolizumab, Durvalumab). The VITALITY study, which primarily employed atezolizumab (PD-L1) in combination with bevacizumab, reported manageable rejection rates compared to earlier studies utilizing nivolumab (PD-1). Mechanistically, blockade of PD-L1 allows the PD-L2/PD-1 interaction to remain intact. PD-L2 serves as another ligand for PD-1, and its continued interaction may preserve a residual pathway for peripheral tolerance, potentially rendering PD-L1 inhibitors safer in the context of transplantation. Additionally, concurrent administration of VEGF inhibitors (bevacizumab) may normalize the immune microenvironment and reduce dendritic cell maturation, thereby further mitigating alloimmunity. However, this hypothesis remains purely mechanistic and exploratory. Currently, no prospective, randomized, or direct comparative clinical data are available to establish a differential safety threshold between PD-L1 and PD-1 inhibitors in the transplant setting. The application of differential washout durations based on drug classes would be premature and potentially harmful. Therefore, washout protocols should be rigorously applied to all drug classes [37].

Pragmatic Recommendation: Based on the synthesis of data from the VITALITY study, the International Cohort (Moeckli), and the meta-analysis (Rezaee-Zavareh), a strongly recommended minimum washout period of 12 weeks (3 months) between the last ICI dose and liver transplantation is advised based on the current best available observational evidence. It is important to acknowledge that this recommendation is derived primarily from retrospective cohort data and individual patient data meta-analyses, which are inherently subject to selection bias and confounding factors. The optimal washout duration may vary depending on individual patient characteristics (including irAE history and performance status), underlying tumor biology, and the specific ICI agent administered. Until prospective randomized data become available, clinical judgment should be applied on a case-by-case basis within a multidisciplinary framework. For patients approaching the top of the waitlist, ICIs should be withheld, and alternative locoregional therapies (TACE, Y90 radioembolization) should be employed to maintain tumor control during the washout period [29, 30, 37].

In the context of Living Donor Liver Transplantation (LDLT), ethical considerations are significantly heightened. Subjecting a recipient to a high risk of ICI-induced graft loss also affects the donor, whose healthy organ may be sacrificed without benefit to the recipient. Therefore, strict adherence to the 12-week washout protocol is ethically imperative in LDLT to prevent the futile use of live donor grafts [37].

## Post-transplant checkpoint inhibition: salvage therapy for recurrence

In contrast to the pre-transplant context, the administration of immune checkpoint inhibitors (ICIs) post-transplantation involves the delivery of an immunostimulatory agent directly to a patient with an established allograft [38–40]. As shown in Table 1B, this constitutes a highly perilous salvage pathway. The first-line standard of care should remain tyrosine kinase inhibitors (TKIs), such as Sorafenib or Lenvatinib, because of their mechanism of vascular endothelial growth factor (VEGF) inhibition, which ensures high graft safety without the risk of rejection, albeit with moderate efficacy. Should TKIs prove ineffective, transitioning to ICI therapy becomes an absolute "Last Resort." Systematic reviews by Abdel-Wahab and d’Izarny-Gargas documented severe allograft rejection risks ranging from 30 to 40% in stable liver transplant recipients treated with ICIs for recurrent cancer [39]. More concerningly, rejection occurring in the post-transplant salvage context is associated with an absolute mortality rate approaching 50%, primarily due to rapid graft failure. The underlying mechanism involves the immediate reactivation of resident memory T-cells (TRM) extensively embedded within graft tissue. Experimental mitigation strategies, such as substituting the primary calcineurin inhibitor with an mTOR inhibitor (e.g., sirolimus), do not ensure allograft safety [42]. Available data from systematic reviews indicate that post-transplant ICI therapy achieves objective response rates of approximately 20–30% in HCC recurrence, although this modest oncological benefit must be carefully weighed against the risk of severe allograft rejection. Patient selection for ICI salvage therapy should prioritize individuals with (1) rapidly progressive HCC recurrence refractory to first-line TKI therapy, (2) stable allograft function with no recent rejection episodes, (3) adequate performance status, and (4) comprehensive informed consent regarding the high risk of graft loss. Regarding timing, the interval between transplantation and ICI initiation may influence rejection risk; early post-transplant administration (within 2 years) is associated with significantly higher rejection rates than late administration (> 2 years), likely reflecting the relative maturity of graft tolerance at later time points. Regarding immunosuppressive management, maintaining tacrolimus trough levels at the upper end of the therapeutic range (10–15 ng/mL) during ICI therapy, combined with close clinical and biochemical surveillance, represents a pragmatic approach in experienced centers [41, 42].

## Disease-specific modalities and expanding indications

While systemic immune checkpoint inhibitors (ICIs) are the standard of care for advanced hepatocellular carcinoma (HCC), their expanding application in the adjuvant setting is currently undergoing rigorous re-evaluation. The IMbrave050 trial initially indicated a benefit in recurrence-free survival; however, updated mature results have shown the convergence of the survival curves at later time points. Prior exposure to these agents may significantly complicate future transplant eligibility because of the requisite 12-week washout period [43].

In advanced biliary tract cancers, the TOPAZ-1 and KEYNOTE-966 trials established combined chemo-immunotherapy as the new global standard [44, 45]. However, in the context of liver transplantation for perihilar cholangiocarcinoma, the systemic administration of these agents is generally considered an absolute contraindication. Similarly, while liver transplantation for colorectal liver metastases (CRLM) is gaining acceptance following the SECA trials, the use of immune checkpoint inhibitors (ICIs) is restricted to the rare microsatellite instability-high (MSI-H) subtype, necessitating adherence to the same 90-day washout principle [46, 47].

## Emerging risk stratification biomarkers

Transplant oncology is swiftly advancing towards precision medicine frameworks that incorporate predictive biomarkers, as depicted in the lower panel of Table 1.

### Pre-transplant irAEs

Fang et al. conducted a comprehensive cohort study that established a significant correlation between a patient’s history of systemic immune activation and subsequent graft rejection. These two biomarkers were selected because they represent complementary dimensions of rejection risk: pre-transplant irAEs serve as a systemic phenotypic indicator of immune hyperactivation, reflecting the degree to which the host immune system has been globally primed by ICI therapy, whereas the Eplet Risk Score provides a genotypic quantification of the HLA molecular mismatch burden, capturing inherent alloimmune susceptibility independent of prior ICI exposure. Patients on the waitlist who experienced severe immune-related adverse events (irAEs), such as immune checkpoint inhibitor (ICI)-induced rash or autoimmune colitis, were found to be 9.17 times more likely to reject the liver allograft post-transplantation. The presence of irAEs serves as a phenotype indicative of a systemically hyperactivated immune state. A documented history of severe irAEs necessitates a considerably extended washout period or implementation of aggressive induction immunosuppression [48].

### The Eplet risk score

Hirata et al. formally introduced the Eplet Risk Score as a method to accurately quantify three-dimensional Human Leukocyte Antigen (HLA) molecular mismatches. This study demonstrated that a high HLA molecular mismatch load significantly contributes to the formation of de novo donor-specific antibodies (DSA). Patients with elevated Eplet Risk Scores are particularly susceptible to immune checkpoint inhibitor (ICI)-induced alloimmunity, as their unrestrained immune systems possess a greater number of potent structural targets to attack once the checkpoint “brakes” are released [49].

### Graft PD-L1 expression as a predictor of post-transplant ICI-induced rejection

Beyond peripheral and HLA-based markers, intragraft PD-L1 immunohistochemistry has emerged as a direct tissue-based predictor of rejection risk in liver transplant recipients receiving post-transplant PD-1 inhibitor therapy, and is summarized as the third predictive biomarker entry in Table 1. Mechanistically, PD-L1 expressed by hepatocytes, cholangiocytes, and sinusoidal endothelial cells engages PD-1 on graft-infiltrating T-cells to dampen alloreactive responses; therefore, pharmacological PD-1 blockade disproportionately disrupts this counter-regulatory pathway in PD-L1–positive grafts, exposing them to T-cell-mediated rejection. In a pilot retrospective study by Shi et al., among five liver transplant recipients receiving anti-PD-1 therapy for HCC recurrence, the single patient with PD-L1–positive graft staining developed acute rejection, whereas all four PD-L1–negative recipients did not, supporting graft PD-L1 as a candidate selection biomarker [50]. This concept was subsequently validated in a single-center prospective single-arm pilot trial by He et al., in which 20 consecutive liver transplant recipients with confirmed pre-treatment PD-L1–negative grafts received PD-1 inhibitor therapy for recurrent or metastatic liver cancer; the observed acute rejection rate was 15% (3/20; 95% CI, 3.2–37.9%), and median post-recurrence survival reached 24.6 months, exceeding historical controls (16.3 months) [51]. Together, these data suggest that pre-treatment graft biopsy with PD-L1 immunostaining may help identify a subset of post-transplant patients in whom PD-1 inhibition can be considered with an acceptable, although not negligible, rejection risk. However, both studies are limited by their small sample sizes, single-center design, and reliance on PD-1 inhibitors only. The predictive value of graft PD-L1 for PD-L1 and CTLA-4 inhibitors remains undefined, and prospective multicenter validation is required before routine clinical implementation.

### Proposed biomarker-guided clinical framework

Based on the currently available evidence, the following pragmatic framework for biomarker-guided decision-making is proposed, pending prospective validation. For patients with a history of Grade ≥ 2 irAEs, an extended washout period (potentially exceeding 6 months) is advisable, accompanied by aggressive immunosuppression induction with anti-thymocyte globulin (ATG) at transplantation. For patients with elevated Eplet Risk Scores, pre-transplant donor selection should aim to minimize HLA molecular mismatch where feasible, and enhanced post-transplant monitoring for de novo DSA should be implemented. For post-transplant candidates being considered for ICI salvage therapy, pre-treatment graft biopsy with PD-L1 immunostaining should be considered, with negative PD-L1 status favoring a more cautious approach to anti-PD-1 therapy. For patients with multiple risk factors, careful consideration should be given to whether transplantation or ICI rechallenge is appropriate in the absence of a completed washout period. This framework requires validation in prospective cohort studies before routine implementation.

## Conclusions and ethical imperatives

The incorporation of immune checkpoint inhibitors (ICIs) into the delicate framework of liver transplantation represents a significantly high-risk, high-reward frontier in contemporary medicine. The pre-transplant application of ICIs is indisputably beneficial, as evidenced by the VITALITY study, which showed that these treatments effectively downstage aggressive hepatocellular carcinoma (HCC), achieving survival rates of 85%. Nonetheless, a strongly recommended washout period exceeding 90 days remains the best currently validated approach, based on observational evidence, to mitigate the substantial risk of fatal hepatic necrosis to the baseline level. The post-transplant use of ICIs as a salvage strategy entails a severe rejection risk of 30–40% and a mortality rate of approximately 50%, thereby confining it to a measure of absolute last resort.

Clinical teams are required to implement predictive stratification tools employing irAE history and Eplet Risk Scores to identify high-risk phenotypes. The ethical landscape of ICI use differs significantly between LDLT and deceased donor liver transplantation (DDLT). In DDLT, the decision to proceed despite the residual ICI pharmacodynamic risk primarily affects the recipient and the allocation of a deceased donor organ within an equitable waitlist framework. However, in LDLT, a healthy living donor undergoes major hepatic resection with an inherent short-term mortality risk of approximately 0.1–0.5%. ICI-induced fatal graft rejection creates a dual ethical burden: the recipient suffers a potentially avoidable iatrogenic catastrophe, and the donor’s surgical sacrifice is rendered futile. Rigorous informed consent processes for LDLT must explicitly address the following: (1) the pharmacodynamic mechanism and duration of ICI activity; (2) the evidence base for and limitations of the recommended 90-day washout threshold; (3) the documented risk of fatal hepatic necrosis in non-adherent cases; and (4) the absence of reliable rescue strategies once severe rejection is established. Formal multidisciplinary ethics consultation should be considered in any case where tumor progression prevents completion of the recommended washout period, as the living donor’s autonomous right to an informed and meaningful contribution must be independently protected, irrespective of the recipient’s clinical urgency. In the context of Living Donor Liver Transplantation (LDLT), strict adherence to the Safe Zone (> 90 days) is an ethical imperative to ensure that the sacrifice of a perfectly healthy living donor does not culminate in a futile outcome due to a predictable iatrogenic immunological catastrophe. Until new safety pathways are validated, time remains the most reliable means of resolving the life-threatening conflict between oncological immunity and allograft tolerance in these patients.

## Data Availability

The data supporting the findings of this study are available from the corresponding author.

## Abbreviations

HCC: Hepatocellular carcinoma
ICI: Immune checkpoint inhibitor
LDLT: Living donor liver transplantation

## References

[1] Mazzaferro V, Regalia E, Doci R et al (1996) Liver transplantation for the treatment of small hepatocellular carcinomas in patients with cirrhosis. N Engl J Med 334(11):693–699 8594428 10.1056/NEJM199603143341104

[2] Hibi T, Itano O, Shinoda M et al (2017) Liver transplantation for hepatobiliary malignancies: a new era of “Transplant Oncology” has begun. Surg Today 47(4):403–415 27130463 10.1007/s00595-016-1337-1

[3] Sapisochin G, Hibi T, Toso C et al (2021) Transplant oncology in primary and metastatic liver tumors: principles, evidence, and opportunities. Ann Surg 273(3):483–493 33065633 10.1097/SLA.0000000000004071

[4] Sharpe AH, Pauken KE (2018) The diverse functions of the PD1 inhibitory pathway. Nat Rev Immunol 18(3):153–167 28990585 10.1038/nri.2017.108

[5] Keir ME, Liang SC, Guleria I et al (2006) Tissue expression of PD-L1 mediates peripheral T cell tolerance. J Exp Med 203(4):883–895 16606670 10.1084/jem.20051776 PMC2118286

[6] Tabrizian P, Florman SS, Schwartz ME (2021) PD-1 inhibitor as bridge therapy to liver transplantation? Am J Transplant 21(5):1979–1980 33316117 10.1111/ajt.16448

[7] Schwacha-Eipper B, Minciuna I, Banz V et al (2020) Immunotherapy as a Downstaging Therapy for Liver Transplantation. Hepatology 72(4):1488–1490 32171041 10.1002/hep.31234

[8] Calne RY, White HJ, Yoffa DE et al (1967) Prolonged survival of liver transplants in the pig. Br Med J 4(5580):645–648 4862477 10.1136/bmj.4.5580.645 PMC1749193

[9] Calne RY, Sells RA, Pena JR et al (1969) Induction of immunological tolerance by porcine liver allografts. Nature 223(5205):472–476 4894426 10.1038/223472a0

[10] Starzl TE, Demetris AJ, Murase N et al (1992) Cell migration, chimerism, and graft acceptance. Lancet 339(8809):1579–1582 1351558 10.1016/0140-6736(92)91840-5 PMC2950640

[11] Starzl TE, Demetris AJ, Trucco M et al (1993) Chimerism after liver transplantation for type IV glycogen storage disease and type 1 Gaucher’s disease. N Engl J Med 328(11):745–749 8437594 10.1056/NEJM199303183281101 PMC2963442

[12] Iwai Y, Ishida M, Tanaka Y et al (2002) Involvement of PD-L1 on tumor cells in the escape from host immune system and tumor immunotherapy by PD-L1 blockade. Proc Natl Acad Sci U S A 99(19):12293–12297 12218188 10.1073/pnas.192461099 PMC129438

[13] Iwai Y, Terawaki S, Ikegawa M et al (2003) PD-1 inhibits antiviral immunity at the effector phase in the liver. J Exp Med 198(1):39–50 12847136 10.1084/jem.20022235 PMC2196084

[14] Latchman YE, Liang SC, Wu Y et al (2004) PD-L1-deficient mice show that PD-L1 on T cells, antigen-presenting cells, and host tissues negatively regulates T cells. Proc Natl Acad Sci U S A 101(29):10691–10696 15249675 10.1073/pnas.0307252101 PMC489996

[15] Probst HC, McCoy K, Malinverni T et al (2005) Resting dendritic cells induce peripheral T cell tolerance via PD-1 and CTLA-4. Nat Immunol 6(3):280–286 15685176 10.1038/ni1165

[16] Riella LV, Paterson AM, Sharpe AH et al (2012) Role of the PD-1 pathway in the immune response. Am J Transplant 12(10):2575–2587 22900886 10.1111/j.1600-6143.2012.04224.x PMC3784243

[17] Mazzaferro V, Bhoori S, Sposito C et al (2011) Milan criteria in liver transplantation for hepatocellular carcinoma: an evidence-based analysis of 15 years of experience. Liver Transpl 17(Suppl 2):S44-57 21695773 10.1002/lt.22365

[18] Yao FY, Ferrell L, Bass NM et al (2001) Liver transplantation for hepatocellular carcinoma: expansion of the tumor size limits does not adversely impact survival. Hepatology 33(6):1394–1403 11391528 10.1053/jhep.2001.24563

[19] Mazzaferro V, Llovet JM, Miceli R et al (2009) Predicting survival after liver transplantation in patients with hepatocellular carcinoma beyond the Milan criteria: a retrospective, exploratory analysis. Lancet Oncol 10(1):35–43 19058754 10.1016/S1470-2045(08)70284-5

[20] Dubay D, Sandroussi C, Sandhu L et al (2011) Liver transplantation for advanced hepatocellular carcinoma using poor tumor differentiation on biopsy as an exclusion criterion. Ann Surg 253(1):166–172 21294289 10.1097/sla.0b013e31820508f1

[21] Shimamura T, Akamatsu N, Fujiyoshi M et al (2019) Expanded living-donor liver transplantation criteria for patients with hepatocellular carcinoma based on the Japanese nationwide survey: The 5-5-500 rule. Transpl Int 32(4):356–368 30556935 10.1111/tri.13391

[22] Duvoux C, Roudot-Thoraval F, Decaens T et al (2012) Liver transplantation for hepatocellular carcinoma: a model including alpha-fetoprotein improves the performance of Milan criteria. Gastroenterology 143(4):986–994 22750200 10.1053/j.gastro.2012.05.052

[23] Llovet JM, Ricci S, Mazzaferro V et al (2008) Sorafenib in advanced hepatocellular carcinoma. N Engl J Med 359(4):378–390 18650514 10.1056/NEJMoa0708857

[24] Kudo M, Finn RS, Qin S et al (2018) Lenvatinib versus sorafenib in first-line treatment of patients with unresectable hepatocellular carcinoma: a randomised phase 3 non-inferiority trial. Lancet 391(10126):1163–1173 29433850 10.1016/S0140-6736(18)30207-1

[25] Finn RS, Qin S, Ikeda M et al (2020) Atezolizumab plus bevacizumab in unresectable hepatocellular carcinoma. N Engl J Med 382(20):1894–1905 32402160 10.1056/NEJMoa1915745

[26] Abou-Alfa GK, Lau G, Kudo M et al (2022) Tremelimumab plus durvalumab in unresectable hepatocellular carcinoma. NEJM Evid. 1(8):2100070. 10.1056/EVIDoa2100070 10.1056/EVIDoa2100070 38319892

[27] Qin S, Chan SL, Gu S et al (2023) Camrelizumab plus rivoceranib versus sorafenib as first-line therapy for unresectable hepatocellular carcinoma (CARES-310): a randomised, open-label, international phase 3 study. Lancet 402(10408):1133–1146 37499670 10.1016/S0140-6736(23)00961-3

[28] Llovet JM, Kudo M et al (2025) Transarterial chemoembolisation combined with lenvatinib plus pembrolizumab versus dual placebo for unresectable, non-metastatic hepatocellular carcinoma (LEAP-012): a multicentre, randomised, double-blind, phase 3 study. Lancet 405(10474):203–215 39798578 10.1016/S0140-6736(24)02575-3

[29] Moeckli B, Wassmer CH, El Hajji S et al (2025) Determining safe washout period for immune checkpoint inhibitors prior to liver transplantation: An international retrospective cohort study. Hepatology 82(5):1122–1137 40042053 10.1097/HEP.0000000000001289

[30] Rezaee-Zavareh MS, Yeo YH, Wang T et al (2025) Impact of pre-transplant immune checkpoint inhibitor use on post-transplant outcomes in HCC: A systematic review and individual patient data meta-analysis. J Hepatol 82(1):107–119 38996924 10.1016/j.jhep.2024.06.042 PMC11655254

[31] Nordness MF, Hamel S, Godfrey CM et al (2020) Fatal hepatic necrosis after nivolumab as a bridge to liver transplant for hepatocellular carcinoma. Am J Transplant 20(3):879–883 31550417 10.1111/ajt.15617 PMC10176099

[32] Schnickel GT, Fabbri K, Hosseini M et al (2022) Liver transplantation for hepatocellular carcinoma following checkpoint inhibitor therapy with nivolumab. Am J Transplant 22(6):1699–1704 35080128 10.1111/ajt.16965 PMC9177653

[33] Dehghan Y, Schnickel GT, Hosseini M et al (2021) Rescue liver re-transplantation after graft loss due to severe rejection in the setting of pre-transplant nivolumab therapy. Clin J Gastroenterol 14(6):1718–1724 34643885 10.1007/s12328-021-01521-4 PMC8557174

[34] Friend BD, Venick RS, McDiarmid SV et al (2017) Fatal orthotopic liver transplant organ rejection induced by a checkpoint inhibitor in two patients with refractory, metastatic hepatocellular carcinoma. Pediatr Blood Cancer. 10.1002/pbc.26682 28643391 10.1002/pbc.26682

[35] Guo Z, Liu Y (2024) LiPre-transplantPretransplant use of immune checkpoint inhibitors for hepatocellular carcinoma: a multicenter, retrospective cohort study. Am J Transplant 24(10):1837–1856 38642712 10.1016/j.ajt.2024.04.007

[36] Qiao ZY, Zhang ZJ, Lv ZC et al (2021) Neoadjuvant programmed cell death 1 (PD-1) inhibitor treatment in patients with hepatocellular carcinoma before liver transplant: A cohort study and literature review. Front Immunol 12:653437. 10.3389/fimmu.2021.653437 34349755 10.3389/fimmu.2021.653437 PMC8326904

[37] Tabrizian P, Holzner ML, Ajmera V et al (2025) Intention-to-treat outcomes of patients with hepatocellular carcinoma receiving immunotherapy before liver transplant: The multicenter VITALITY study. J Hepatol 82(3):512–522 39255928 10.1016/j.jhep.2024.09.003

[38] Abdel-Wahab N, Safa H, Abudayyeh A et al (2019) Checkpoint inhibitor therapy for cancer in solid organ transplantation recipients: an institutional experience and systematic review of the literature. J Immunother Cancer 7(1):106. 10.1186/s40425-019-0585-1 30992053 10.1186/s40425-019-0585-1 PMC6469201

[39] d’Izarny-Gargas T, Durrbach A, Zaidan M (2020) Efficacy and tolerance of immune checkpoint inhibitors in solid organ transplant patients with cancer: a systematic review. Am J Transplant 20(9):2457–2465 32027461 10.1111/ajt.15811

[40] DeLeon TT, Salomao MA, Aqel BA et al (2018) Pilot evaluation of PD-1 inhibition in metastatic cancer patients with a history of liver transplantation: the Mayo Clinic experience. J Gastrointest Oncol 9(6):1054–1062 30603124 10.21037/jgo.2018.07.05 PMC6286929

[41] Spain L, Higgins R, Gopalakrishnan K et al (2016) Acute renal allograft rejection after immune checkpoint inhibitor therapy for metastatic melanoma. Ann Oncol 27(6):1135–1137 26951628 10.1093/annonc/mdw130

[42] Esfahani K, Al-Aubodah O, Thebault P et al (2019) Targeting the mTOR pathway uncouples the efficacy and toxicity of PD-1 blockade in renal transplantation. Nat Commun 10:4712. 10.1038/s41467-019-12628-1 31624262 10.1038/s41467-019-12628-1 PMC6797722

[43] Qin S, Chen M, Cheng AL et al (2023) Atezolizumab plus bevacizumab versus active surveillance in patients with resected or ablated high-risk hepatocellular carcinoma (IMbrave050): a randomised, open-label, multicentre, phase 3 trial. Lancet 402(10415):1835–1847 37871608 10.1016/S0140-6736(23)01796-8

[44] Oh DY, Ruth He A, Qin S et al (2022) Durvalumab plus gemcitabine and cisplatin in advanced biliary tract cancer. NEJM Evid. 1(8):2200015. 10.1056/EVIDoa2200015 10.1056/EVIDoa2200015 38319896

[45] Kelley RK, Ueno M, Yoo C et al (2023) Pembrolizumab in combination with gemcitabine and cisplatin compared with gemcitabine and cisplatin alone for patients with advanced biliary tract cancer (KEYNOTE-966): a randomised, double-blind, placebo-controlled, phase 3 trial. Lancet 401(10391):1853–1865 37075781 10.1016/S0140-6736(23)00727-4

[46] Hagness M, Foss A, Line PD et al (2013) Liver transplantation for nonresectable liver metastases from colorectal cancer. Ann Surg 257(5):800–806 23360920 10.1097/SLA.0b013e3182823957

[47] Line PD, Hagness M, Berstad AE et al (2015) A novel concept for partial liver transplantation in nonresectable colorectal liver metastases: The RAPID Concept. Ann Surg 262(1):e5-9 25692361 10.1097/SLA.0000000000001165

[48] Fang J, Zhong S, Wang T et al (2025) Immune-related adverse events are a potent predictor of post-transplant rejection in HCC: a multicentre retrospective cohort study. Gut 4:336719. 10.1136/gutjnl-2025-336719 10.1136/gutjnl-2025-336719 PMC13217134 41193174

[49] Hirata M, Tsukita K, Shindo T et al (2025) Cross-organ hierarchy of HLA molecular mismatches in donor-specific antibody development in solid organ transplantations. Cell Rep Med 6(6):102153. 10.1016/j.xcrm.2025.102153 40449481 10.1016/j.xcrm.2025.102153 PMC12208338

[50] Shi GM, Wang J, Huang XW et al (2021) Graft programmed death ligand 1 expression as a marker for transplant rejection following anti-programmed death 1 immunotherapy for recurrent liver tumors. Liver Transpl 27(3):444–449. 10.1002/lt.25887 32897657 10.1002/lt.25887

[51] He Y, Huang X, Huang X et al (2026) Graft PD-L1 as a predictive marker for rejection in PD-1 inhibitor therapy for recurrent liver tumors after transplant: A prospective pilot trial. Liver Transpl 32(2):135–143. 10.1097/LVT.0000000000000719 40879506 10.1097/LVT.0000000000000719

